# Supplementary material for: Non-alcoholic fatty liver disease is not associated with impairment in health-related quality of life in virally suppressed persons with human immune deficiency virus
Source: PLoS One. 2023 Feb 10;18(2):e0279685. doi: 10.1371/journal.pone.0279685 (PMC9916563; doi:10.1371/journal.pone.0279685)
Supplement: S1 Table — (DOCX) [file pone.0279685.s001.docx]

**Supplementary Table 1. Factors associated with health-related quality of life in persons with HIV based on univariate analysis**

| **Variable** | **PCS** | | | **MCS** | | |
| --- | --- | --- | --- | --- | --- | --- |
|  | **β estimate** | **SE** | **P-value** | **β estimate** | **SE** | **P-value** |
| NAFLD (ref = No NAFLD) | 0.74 | 1.56 | 0.64 | 1.22 | 1.73 | 0.48 |
| NAFLD with CSF (ref = NAFLD without CSF) | -4.46 | 3.20 | 0.16 | 2.66 | 3.90 | 0.49 |
| Age | -0.17 | 0.06 | 0.01 | 0.20 | 0.07 | <0.01 |
| BMI | -0.31 | 0.13 | 0.02 | 0.41 | 0.14 | 0.01 |
| Gender |  |  |  |  |  |  |
| Female | Reference | | | Reference | | |
| Male | 2.86 | 1.93 | 0.14 | -2.27 | 2.15 | 0.29 |
| Transgender female | 6.99 | 3.58 | 0.05 | 3.27 | 3.99 | 0.41 |
| Race |  |  |  |  |  |  |
| White | Reference | | | Reference | | |
| Black | -4.51 | 1.64 | 0.01 | -4.06 | 1.82 | 0.03 |
| Other | 1.36 | 2.81 | 0.63 | 4.81 | 3.11 | 0.12 |
| Ethnicity |  |  |  |  |  |  |
| Hispanic or Latino | Reference | | | Reference | | |
| Non-Hispanic or Latino | -2.84 | 1.68 | 0.09 | -8.43 | 1.79 | <0.01 |
| Waist circumference | -0.20 | 0.05 | <0.01 | 0.16 | 0.05 | <0.01 |
| Diabetes |  |  |  |  |  |  |
| Yes | -8.03 | 2.33 | <0.01 | -1.51 | 2.66 | 0.57 |
| No | Reference | | | Reference | | |
| ALT | 0.05 | 0.04 | 0.24 | 0.02 | 0.04 | 0.61 |
| AST | 0.05 | 0.06 | 0.38 | 0.05 | 0.06 | 0.40 |
| Platelet | -0.01 | 0.01 | 0.46 | -0.00 | 0.01 | 0.77 |
| Triglycerides | -0.01 | 0.01 | 0.04 | -0.02 | 0.01 | 0.03 |
| Fasting glucose | -0.07 | 0.04 | 0.07 | -0.05 | 0.05 | 0.32 |
| Insulin | -0.04 | 0.02 | 0.07 | -0.04 | 0.02 | 0.12 |
| Absolute CD4 | 0.00 | 0.00 | 0.75 | -0.01 | 0.00 | 0.04 |
| Nadir CD4 | 0.01 | 0.01 | 0.16 | -0.01 | 0.01 | 0.04 |

PCS: physical component summary score, MCS: mental component summary score, CSF: clinically significant fibrosis
