# Supplementary material for: Non-alcoholic fatty liver disease is not associated with impairment in health-related quality of life in virally suppressed persons with human immune deficiency virus
Source: PLoS One. 2023 Feb 10;18(2):e0279685. doi: 10.1371/journal.pone.0279685 (PMC9916563; doi:10.1371/journal.pone.0279685)
Supplement: S2 Table — (DOCX) [file pone.0279685.s002.docx]

**Supplementary Table 2. Comparison of subjects’ characteristics between primary NAFLD and HIV-NAFLD**

| **Variable** |  | **HIV-NAFLD**  **(n = 87*)** | **Primary NAFLD (n = 474)** | **P-value** |
| --- | --- | --- | --- | --- |
| Age Mean (SD) |  | 51.8 (10.6) | 52.9 (12.9) | 0.38 |
| Sex |  |  |  | <0.01 |
| Male |  | 71 (81.6%) | 180 (38%) |  |
| Female |  | 16 (18.4%) | 293 (62%) |  |
| Race |  |  |  | <0.01 |
| White |  | 62 (71.3%) | 447 (94.3%) |  |
| Black |  | 19 (21.8%) | 7 (1.5%) |  |
| Other** |  | 6 (6.9%) | 20 (4.2%) |  |
| Ethnicity |  |  |  | <0.01 |
| Hispanic or Latino |  | 26 (29.9%) | 6 (1.3%) |  |
| Non-Hispanic or Latino |  | 60 (69%) | 333 (70.2%) |  |
| Refused/Unknown |  | 1 (1.2%) | 135 (28.5%) |  |
| BMI Mean (SD) |  | 30.49 (5.7) | 35.78 (7.4) | <0.01 |
| Obesity |  | 39 (44.8%) | 375 (79.3%) | <0.01 |
| Diabetes |  | 15 (17.4%) | 180 (38%) | <0.01 |
| HTN |  | 33 (37.9%) | 202 (42.6%) | 0.42 |
| ALT Mean (SD) |  | 37.8 (22.6) | 36.8 (21.5) | 0.70 |
| AST Mean (SD) |  | 30.2 (16.1) | 35.0 (18.7) | 0.03 |
| Platelets Mean (SD) |  | 237.5 (66.7) | 231.6 (87.6) | 0.50 |
| Triglycerides Mean (SD) |  | 177.7 (149.7) | 168.0 (84.9) | 0.59 |
| Fasting glucose Mean (SD) |  | 109.8 (69.0) | 120.9 (52.0) | 0.43 |
| Insulin Mean (SD) |  | 30.0 (6.6) | 26.0 (27.4) | 0.72 |
| LSM$\geq$8.6 kPa |  | 11 (12.6%) | 214 (45.2%) | <0.01 |

* Transgender females with HIV were excluded from this analysis, ** Other: combined other race categories with very few frequencies including Asians, native Hawaiian, American Indians and Unknown races
