# Supplementary material for: Non-alcoholic fatty liver disease is not associated with impairment in health-related quality of life in virally suppressed persons with human immune deficiency virus
Source: PLoS One. 2023 Feb 10;18(2):e0279685. doi: 10.1371/journal.pone.0279685 (PMC9916563; doi:10.1371/journal.pone.0279685)
Supplement: S3 Table — (DOCX) [file pone.0279685.s003.docx]

**Supplementary Table 3. Factors associated with physical and mental components of health-related quality of life in patients with NAFLD in univariate analysis**

|  | **PCS** | | | **MCS** | | |
| --- | --- | --- | --- | --- | --- | --- |
| **Variable** | **β estimate** | **SE** | **P-value** | **β estimate** | **SE** | **P-value** |
| Group (ref = Primary NAFLD) |  |  |  |  |  |  |
| HIV-NAFLD | 7.61 | 1.37 | <0.01 | 2.34 | 1.32 | 0.07 |
| CSF (ref = No CSF) | -6.26 | 1.00 | <0.01 | -1.27 | 0.97 | 0.19 |
| Age | -0.22 | 0.04 | <0.01 | 0.19 | 0.04 | <0.01 |
| Sex |  |  |  |  |  |  |
| Female | Reference | | | Reference | | |
| Male | 5.49 | 1.00 | <0.01 | 3.81 | 0.95 | <0.01 |
| Race |  |  |  |  |  |  |
| White | Reference | | | Reference | | |
| Black | 3.53 | 2.40 | 0.14 | -2.13 | 2.27 | 0.35 |
| Other | 7.15 | 2.40 | <0.01 | 2.17 | 2.27 | 0.34 |
| Ethnicity |  |  |  |  |  |  |
| Non-Hispanic or Latino | Reference | | | Reference | | |
| Hispanic or Latino | 5.03 | 2.19 | 0.02 | 9.22 | 2.04 | <0.01 |
| Refused/Unknown | -2.77 | 1.19 | 0.02 | -0.42 | 1.11 | 0.7 |
| BMI | -0.47 | 0.07 | <0.01 | -0.11 | 0.07 | 0.08 |
| Diabetes |  |  |  |  |  |  |
| Yes | -6.44 | 1.03 | <0.01 | -1.39 | 1.00 | 0.17 |
| No | Reference | | | Reference | | |
| ALT | 0.05 | 0.03 | 0.05 | 0.00 | 0.02 | 0.87 |
| AST | 0.03 | 0.03 | 0.30 | 0.01 | 0.03 | 0.63 |
| Platelet | 0.02 | 0.01 | 0.01 | -0.01 | 0.01 | 0.03 |
| Triglycerides | -0.01 | 0.01 | 0.02 | -0.02 | 0.01 | <0.01 |
| Fasting glucose | -0.07 | 0.01 | <0.01 | -0.03 | 0.01 | 0.03 |
| Insulin | -0.05 | 0.02 | 0.06 | -0.08 | 0.03 | 0.01 |

CSF: Clinically significant fibrosis (defined as LSM ≥ 8.6 kPa)
